# Planning of Ballistic Movement following Stroke: Insights from the Startle Reflex

**DOI:** 10.1371/journal.pone.0043097

**Published:** 2012-08-30

**Authors:** Claire Fletcher Honeycutt, Eric Jon Perreault

**Affiliations:** 1 Sensory Motor Performance Program, Rehabilitation Institute of Chicago, Chicago, Illinois, United States of America; 2 Department of Electrical Engineering and Computer Science, Northwestern University, Chicago, Illinois, United States of America; 3 Department of Biomedical Engineering, Northwestern University, Evanston, Illinois, United States of America; 4 Department of Physical Medicine and Rehabilitation, Northwestern University, Chicago, Illinois, United States of America; Centre Hospitalier Universitaire Vaudois Lausanne - CHUV, UNIL, Switzerland

## Abstract

Following stroke, reaching movements are slow, segmented, and variable. It is unclear if these deficits result from a poorly constructed movement plan or an inability to voluntarily execute an appropriate plan. The acoustic startle reflex provides a means to initiate a motor plan involuntarily. In the presence of a movement plan, startling acoustic stimulus triggers non-voluntary early execution of planned movement, a phenomenon known as the startReact response. In unimpaired individuals, the startReact response is identical to a voluntarily initiated movement, except that it is elicited 30–40 ms. As the startReact response is thought to be mediated by brainstem pathways, we *hypothesized* that the startReact response is intact in stroke subjects. If startReact is intact, it may be possible to elicit more task-appropriate patterns of muscle activation than can be elicited voluntarily. We found that startReact responses were intact following stroke. Responses were initiated as rapidly as those in unimpaired subjects, and with muscle coordination patterns resembling those seen during unimpaired volitional movements. Results were striking for elbow flexion movements, which demonstrated no significant differences between the startReact responses elicited in our stroke and unimpaired subject groups. The results during planned extension movements were less straightforward for stroke subjects, since the startReact response exhibited task inappropriate activity in the flexors. This inappropriate activity diminished over time. This adaptation suggests that the inappropriate activity was transient in nature and not related to the underlying movement plan. We hypothesize that the task-inappropriate flexor activity during extension results from an inability to suppress the classic startle reflex, which primarily influences flexor muscles and adapts rapidly with successive stimuli. These results indicate that stroke subjects are capable of planning ballistic elbow movements, and that when these planned movements are involuntarily executed they can be as rapid and appropriate as those in unimpaired individuals.

## Introduction

Eighty percent of stroke survivors have difficulty executing reaching tasks due in part to muscle weakness and abnormal patterns of muscle activation which arise from impaired volitional and reflex pathways. These impairments lead to reaching movements that are slow, segmented, variable, and restricted in range relative to unimpaired individuals [1], [2]. Goal directed reaching has two distinct stages: planning and execution [3]. Presently, there is little knowledge about the how stroke impacts either stage of movement. It is unclear if the deficits following stroke are due to an inability to voluntarily execute an appropriately planned movement or conversely, due to voluntarily executing a poorly constructed movement plan.

The acoustic startle reflex provides a means to evaluate a planned movement in isolation from voluntary execution. The effect of a startling acoustic stimulus is different dependent on the state of planning. In the absence of a movement plan, startling stimuli evoke an involuntary quick, synchronous burst of muscle activity seen predominately in the flexor muscles – referred to as the classic startle reflex. Subjects adapt to classic startle rapidly, so the effect is seen only in response to the first few stimuli [4]. Alternatively, in the presence of a movement plan, a startling acoustic stimulus triggers an involuntary early initiation and execution of the planned movement, a phenomenon often called the *startReact* response [5], [6]. In unimpaired individuals, startReact initiated movements are analogous to voluntarily initiated movements resulting in comparable patterns of muscle activity and target accuracy. While startReact movements are sometimes reported to be more forceful [7] or faster in velocity [8],the most striking difference is that startReact movements are initiated 30–40 ms faster than voluntary movements. Importantly, startReact does not appear to adapt and can be triggered many times, making it more accessible for study than the classic startle reflex [9].

The classic startle reflex remains intact following stroke, and is often enhanced relative to unimpaired subjects [10]. However, very little is known about the startReact phenomenon in this population. In a review chapter on the startle reflex, Rothwell et al. provided initial evidence suggesting that startReact can be used to increase appropriate muscle activity following stroke [11], but we are unaware of any published work exploring this possibility more thoroughly. Our primary *objective* was to quantify the behavior of the startReact phenomenon in stroke subjects. Our experiments focus on elbow movements to address the often-reported impairments in arm control following stroke (American Heart Association 2009).

Based on the preliminary observations reported in the literature, we *hypothesized* that the startReact response remains intact in stroke subjects, and that it can be used to elicit more task-appropriate patterns of muscle activation than can be elicited voluntarily. Such a finding would suggest that stroke subjects retain the capacity to appropriately plan ballistic motor tasks, but that the lesion interrupts appropriate voluntary execution of that plan. Further, this finding would indicate that the brainstem pathways responsible for triggering startReact remain intact and are potential targets for rehabilitation therapies.

## Materials and Methods

### Subjects

Data were collected from 10 chronic cortical stroke subjects ranging in age from 47–81 (mean: 66+/−9.1) and 10 unimpaired subjects with overlapping age ranges 42–80 (mean: 56+/−12). A slight difference was found in the means of these groups (p = 0.048). However, no age related differences were observed in EMG amplitudes or onset latencies within the unimpaired population. Therefore, all subjects were included. Stroke subjects with a range of impairment levels were recruited (Table 1). Impairment was assessed using the upper extremity Fugl-Meyer score, which ranged from 10–59. Inclusion criteria for the stroke subjects included: a unilateral brain lesion from a stroke at least one year prior to the study, an ability to understand the task, lack of aphasia, and a stroke that affected the arm which was dominant prior to injury were included. We evaluated the dominant arm of all subjects as startReact movements have been studied largely in the dominant arm [12].

**Table 1:** 

*Stroke Subjects*						*Unimpaired Subjects*			
Subject #	Sex	Age	Paretic Limb*	Years since Stroke	FM	Subject #	Sex	Age	Dominant Hand
1	M	68	L	10	34	1	M	63	R
2	M	61	R	8	54	2	M	59	R
3	M	81	R	17	24	3	M	42	L
4	M	72	R	5	56	4	M	60	R
5	M	69	R	6	12	5	M	45	R
6	M	61	R	18	10	6	F	70	R
7	M	65	R	11	22	7	F	51	R
8	M	74	R	23	15	8	M	49	R
9	F	70	R	22	40	9	M	48	R
10	M	47	R	36	59	10	F	80	R

* Paretic limb was dominant hand prior to stroke.

Fugl-Meyer (FM) scores range from 0 (severe impairment) to 66 (mild impairment).

### Ethics Statement

All protocols and recruitment procedures were approved by Northwestern's Institutional Review Board (IRB) under study STU00009204. Informed, written consent was obtained from all subjects and all collected data were de-indentified. Before participating, all subjects were required to communicate their understanding of the facts, implications, and future consequences of the performed research. If subjects were unable to sign their name on the consent form a primary caregiver or guardian was required to be present. Subjects (unimpaired and stroke) were not included or excluded based on socioeconomic status, race, ethnicity, or environmental exposure.

### Equipment

**Figure 1:**
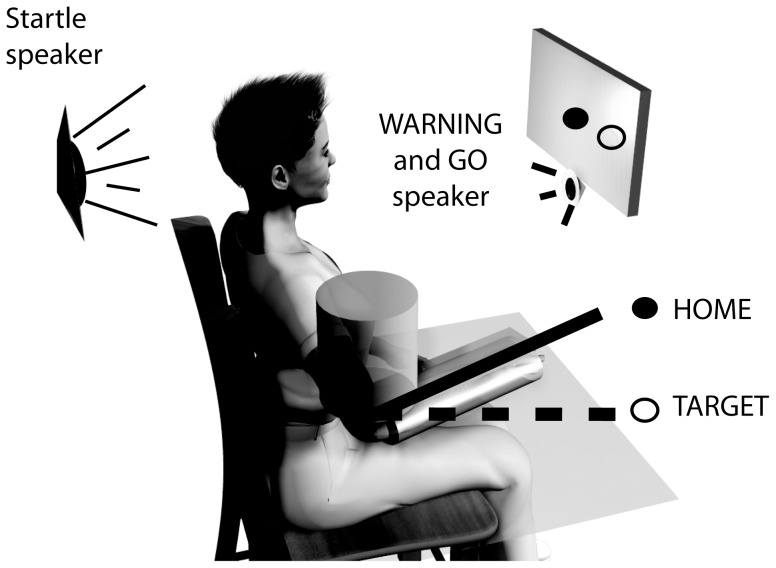
Experimental setup. Subjects were attached to a rotary motor used to support the arm and provide measures of elbow joint angle. Visual feedback was provided on an LCD display to facilitate the completion of the experimental reaching tasks between the Home and Target positions. Auditory cues were provided by a speaker placed in front of the subject. Startling stimuli were delivered by a loudspeaker placed directly behind the subject. Further details are in text.

The experimental setup was designed so that all subjects could complete the elbow movement tasks, even those with low Fugl-Meyer scores. Therefore our set-up restricted arm movement to the elbow and fully supported the arm against gravity.. The latter condition in particular, has been shown to greatly increase the voluntary range of motion for highly impaired subjects [13]. Although these experimental objectives could be obtained using a passively instrumented device, we used an existing motor which had all necessary capabilities. The apparatus consisted of a one degree of freedom rotary motor (BSM90N; Baldor Electric Company, WV), connected to a 10∶1 planetary gear (AD140-010-PO; Apex Dynamics, Taiwan). The motor encoder, coupled with the planetary gear, provided an angular measurement resolution of 3.6×10^−3^ deg. All subjects were connected to the motor using a custom made thermoplastic cast that immobilized the wrist (Fig. 1). A force transducer (45E15A4; JR3 Inc, Woodland, CA) was positioned between the cast and the crank arm of the motor, and was used as part of the motor control system. The center of rotation for the motor was located above the elbow joint which isolated all movements to the elbow flexion/extension axis. The rotary motor did not assist or perturb the elbow in any way. Rather, it was configured as an admittance controller set to mimic the properties of a passive inertial load (0.2 kg-m^2^/rad) in the flexion/extension axis.

Bipolar Ag/AgCl electromyography (EMG) electrodes (Noraxon Dual Electrodes, #272, Noraxon USA Inc., AZ) were used to record muscle activity from the brachioradialis (Br), triceps long head (TriLo), and the left and right sternocleidomastoid (SCML and SCMR) muscles. The biceps brachii muscle is a poor elbow flexor when the forearm is pronated, as in our studies [14], [15]. As a result, the elbow flexor brachioradialis (Br) muscle was chosen. EMG signals were amplified and conditioned using a Bortec AMT-8 (Bortec Biomedical Ltd., Canada), with a band-pass filter of 10–1,000 Hz. The resulting signals were anti-alias filtered using 5^th^ order Bessel filters with a 500 Hz cut-off frequency and sampled at 2500 Hz (PCI-DAS1602/16; Measurement Computing, MA). Elbow position was recorded by an encoder with an effective resolution of 0.0036 degrees. EMG and position data were sampled synchronously. All data collection was synchronized to the GO signal using a pre-trigger collection time of 1 s and post-trigger collection time of 5 s.

### Protocol

In all experiments, the arm was positioned at approximately 70 degrees of shoulder abduction and 25 degrees shoulder flexion; the elbow was positioned at 90 degrees. Subjects were seated in an adjustable, immobile chair. Straps were used to constrain the trunk and shoulder so that only movements of the elbow were possible. Each subject was asked to perform ballistic elbow extension and flexion movements of 25 degrees. Movement order was randomly assigned across subjects. All trials in a particular direction (flexion or extension) were completed before switching to the opposing direction. A computer screen displayed two circles: HOME (located centrally) and TARGET (located at either 25 degrees elbow extension or flexion).

Subjects were instructed to move into the HOME circle and wait for two non-startling (80 dB) auditory cues. The first cue (WARNING) signaled the start of the trial, and indicated that the subject should prepare to move. The second cue (GO) was the prompt to initiate the intended movement as rapidly as possible. The GO occurred randomly 2.2–2.5 seconds following the WARNING consistent with previous startReact experiments [16]. Subjects were asked to move as quickly as possible from the HOME to TARGET circle. Subjects were instructed to attempt to achieve the target for 2–3 seconds before returning to HOME. There was no instruction given for how to respond to the loud acoustic stimulus. Subjects were first trained in the task until they responded reliably and consistently to the GO signal; this typically occurred after approximately 30 trials.

After training, subjects experienced blocks of 15 trials. During each block 3–4 trials were randomly selected to be either startle or startReact trials. For startle trials (startle without a movement plan), the WARNING was replaced with a startling acoustic stimulus of 128 dB. In this scenario, subjects would be in the HOME circle but not prepared to move. For the startReact trials (startle with a movement plan), the GO was replaced with a startling acoustic stimulu**s**. In this scenario, subjects would be in the HOME circle prepared to execute a ballistic movement. Subjects were given no instruction on how to respond to the startling acoustic stimulus. Six to ten startReact trials were collected along with at least two startle trials from each subject. The one exception was stroke subject 1, from which startle trials were not collected. Ballistic elbow movements were selected so that a diverse patient population, including severely impaired and spastic patients could perform the task, and to be consistent with previous studies of the startReact phenomenon.

### Data analysis

Position and EMG traces were visually inspected to eliminate trials in which the subject moved out of HOME prior to the GO or any especially slow trials in which subjects did not move at the GO. Next, SCM muscle activity was evaluated in all trials (voluntary and startReact). Activity in the SCM muscle is known to indicate the presence startle [5], [12]. We considered activity in either the left or right SCM muscles within 150 ms of the acoustic stimulus to indicate the presence of startle [17]. Using this criterion, all trials with SCM activity present in either left or right SCM were classified as SCM+ (startle occurred). Those without SCM activity were classified as SCM- (startle was not detected). Only SCM- voluntary reaching trials and SCM+ startReact reaching trials were analyzed further, which yielded an inclusion rate of 89% voluntary reach and 80% startReact trials in stroke subjects and 93% and 64% respectively in unimpaired subjects. While it is possible to have a startling reaction without the presence of SCM, we wished to only evaluate those trials where startle was definitively present.

The latency of muscle activity onset was calculated for each trial. EMG data were demeaned and rectified. The average background activity and standard deviation were calculated. Next an automated program identified the time at which the processed EMG increased above 2.5 times the standard deviation of the background activity for a period of 15 ms. Following the automatic detection of EMG onset, each trial was evaluated visually to ensure accuracy. The amplitude of the EMG was computed as the average rectified response for a window of 70 ms following the detected onset.

### Statistical Analysis

Our primary hypothesis was that the startReact response remains intact following stroke. This was tested using linear mixed-effect models with group (stroke or unimpaired), movement direction (flexion or extension) and condition (voluntary movement or startReact) as the independent factors. Two separate dependent factors were considered: onset latency and amplitude of the measured EMG. Separate analyses were conducted for each of the recorded muscles. In all analyses, subjects were treated as a random factor to account for the variability associated with sampling a small subset of the unimpaired and stroke populations.

We conducted two additional analyses in addition to testing our primary hypothesis. First, we evaluated how onset latency of the agonistic muscles varied between the most impaired (Fugl-Meyer<20) and less impaired (Fugl-Meyer>20) stroke subjects. This was conducted using an independent, two-tailed t-test. Second, we tested if there was a difference in the mean onset latency between startle, startReact flexion, and startReact extension conditions. Again, only SCM+ trials were considered. A linear mixed-effect model using muscle type (Br or TriLo) and trial type (startle, startReact flexion, startReact extension) as independent factors, and latency as the dependent factor was utilized. Subjects were treated as a random effect.

In accordance with recent standardization of statistical practices, all individual trials were included in our analysis of the linear mixed-effects models [18]. This method has been shown to be more rigorous and powerful than using a single mean for each subject. The use of all trials allows more independent information than a single measurement decreasing the probability of statistical error by capturing all the variability within a data set. Additionally, the mixed-effects models take into account the number of trials in the ANOVA analysis ensuring that data are not misrepresented or inflated due to differences in trial number across subjects in unbalanced data sets. Tukey's Honestly Significant Difference (TukeyHSD), which corrects for multiple comparisons, was used for all *post hoc* comparisons. All statistical analyses were performed using R (R Development Core Team, 2006). All statistical tests were made at a significance level of p<0.05. P-values are noted in main text when they are not otherwise depicted in Figures. All error bars correspond to standard deviations. All statistical measures were completed and verified with an independent statistician.

## Results

### Voluntary and startReact during flexion trials

**Figure 2:**
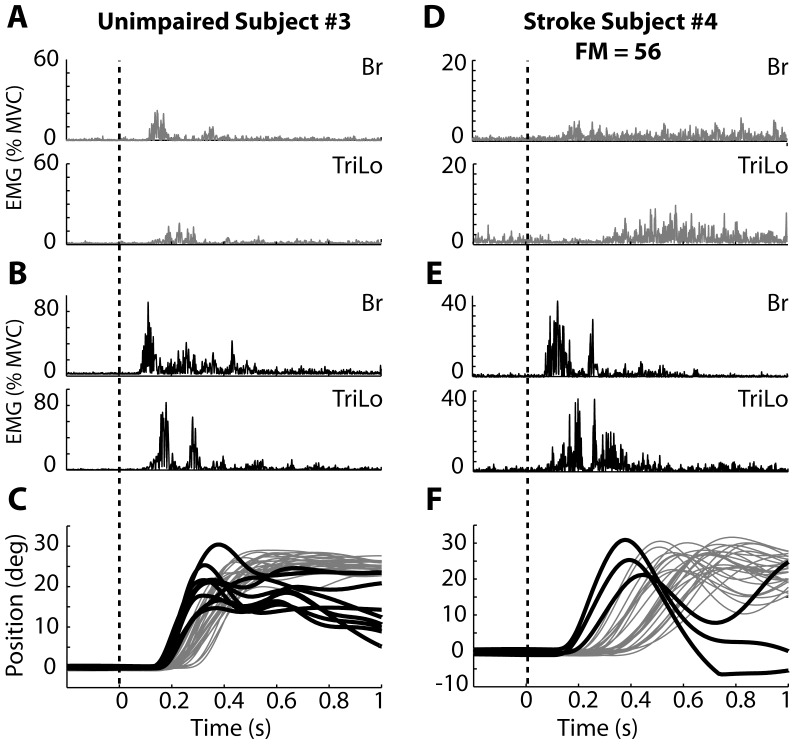
Sample data from typical unimpaired and stroke subjects during flexion. (A, B, C) Data from unimpaired subject. (D, E, F) Data from stroke subject. (A, D) EMG responses in the Br and TriLo muscles during voluntary reaching. (B, E) EMG responses during startReact reaching. (C,F) Elbow position during voluntary (gray) and startReact (black) reaching.

Elbow flexion elicited by the startReact response in unimpaired subjects was initiated faster than voluntary flexion. It also had appropriate patterns of muscle activity, and increased muscle activation. During voluntary flexion of the elbow (Figure 2A), the agonist muscle (Br) was activated prior to the antagonist muscle (TriLo). When the GO cue was replaced by a startling acoustic stimulus to induce a startReact response, activity in both muscles was initiated faster than during the voluntary conditions (Figure 2B). Despite the faster initiation, the general patterns of muscle activity remained consistent with the Br muscle activated prior to the TriLo muscle. The rapid muscle activations during startReact resulted in faster movement initiation, as measured by the change in elbow position (Figure 2C). Finally, muscle activity was increased during startReact flexion.

**Figure 3:**
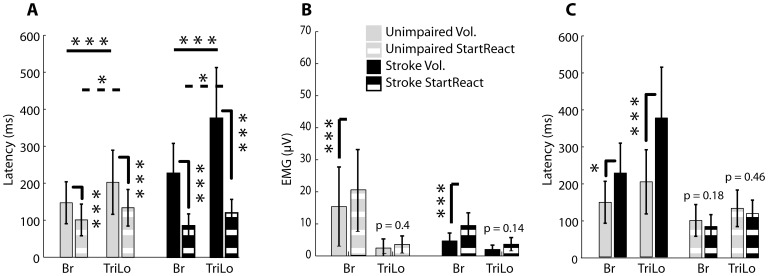
Group **Results** **for flexion task.** (A) Comparison of voluntary and startReact reaching latencies in unimpaired and stroke subjects. (B) Comparison of voluntary and startReact mean EMG amplitudes in unimpaired and stroke subjects. (C) Comparison between reaching latencies of unimpaired and stroke subjects during voluntary and startReact reaching. Same data are presented as in (A), reorganized to facilitate comparison. Means and standard deviations are presented above. Stars indicate significance: * = p-value<.05, ** = p-value<.01, and *** = p-value<.001.

These results were consistent across all unimpaired subjects, as assessed using the analyses described in the Methods. Muscle onset latency was significantly affected by condition (voluntary vs startReact: F_(1,525)_ = 72.96, p<0.0001) and muscle type (Br vs TriLo: F_(1,525)_ = 94.84, p<0.0001); the interaction between these factors (F_(1,525)_ = 5.77, p = 0.0166) was also significant. The onset latency for both muscles was faster during startReact elbow flexion than voluntary elbow flexion (Figure 3A, gray bars). The average voluntary onset latency of unimpaired subjects in the Br muscle was 147+/−56 ms compared to 101+/−42 ms during startReact flexion, a significant difference (p<0.0001). The TriLo muscle was activated at 203+/−86 ms during voluntary trials compared to 134+/−49 ms during startReact flexion, also a significant difference (p<0.0001). Appropriate patterns of muscle activity, with the agonist leading the antagonist, occurred during both startReact (p = 0.03) and voluntary (p<0.0001) conditions. These same factors and their interaction also significantly influenced EMG amplitude (condition: F_(1,524)_ = 20.86, p<0.0001; muscle type: F_(1,524)_ = 425.56, p<0.0001; interaction: F_(1,524)_ = 7.09, p = 0.008). Muscle activity, as assessed by the amplitude of the EMG, was greater for the Br muscle during startReact flexion (20+/−12.8 µV) than voluntary flexion (15+/−12.3 µV) (Figure 3B, gray bars, p<0.0001). While there was also a small increase for the TriLo muscle, it was not statistically significant (p = 0.4).

Similar to unimpaired subjects, elbow flexion movements initiated by the startReact response in stroke subjects were initiated faster, had appropriate patterns of muscle activity, and exhibited increased muscle activation. Voluntary flexion following stroke (Figure 2D) was slower than in unimpaired subjects. Muscle activity in the agonist (Br) led the antagonist (TriLo), but an exaggerated delay between agonist/antagonist firing occurred most subjects. StartReact flexion resulted in faster initiation of agonist and antagonist muscle activity in all subjects (Figure 2E). Despite the faster initiation, the patterns of muscle activity (Br muscle leading TriLo muscle) remained appropriate in the startReact trials. The delay between agonist/antagonist firing, seen in voluntary flexion of stroke subjects, was diminished and appeared similar the delay seen in unimpaired subjects during startReact flexion. As with the unimpaired subjects, the more rapid muscle activations of stroke subjects during the startReact conditions resulted in faster movement initiation measured by the change in elbow position (Figure 2F).

These observations were consistent across stroke subjects. The experimental factors of condition (F_(1,444)_ = 324.06, p<0.0001), muscle (F_(1,444)_ = 222.27, p<0.0001) and their interaction (F_(1,444)_ = 20.08, p<0.0001) all influenced EMG onset latency. The onset latency for both muscles was significantly faster during startReact trials compared to voluntary trials (Figure 3A, black bars). Across all stroke subjects, the average Br muscle onset latency was 226+/−80 ms during voluntary flexion compared to 84+/−32 ms during startReact flexion, a significant difference (p<0.0001). The TriLo muscle was activated at 375+/−137 ms during voluntary flexion and at 119+/−36 ms during startReact flexion, a significant difference (p<0.0001). Muscle activity in the agonist appropriately led that in the antagonist in both startReact (p = 0.01) and voluntary (p<0.0001) conditions (Figure 3A). These same factors influenced EMG amplitude (condition: F_(1,437)_ = 129.81, p<0.0001; muscle type: F_(1,437)_ = 170.65, p<0.0001; interaction: F(_1,437)_ = 33.72, p<0.0001). EMG amplitude was significantly increased in the Br muscle during startReact flexion (9.5+/−3.9 µV) compared to voluntary flexion (4.7+/−2.4 µV) (p<0.0001). Again the TriLo muscle amplitude was not increased (p = 0.14) during startReact flexion (Figure 3B, black bars).

Though voluntary flexion movements of stroke subjects were slower than unimpaired subjects and exhibited agonist/antagonist delays, there were no differences found between these populations during startReact flexion (Figure 3C). During voluntary flexion trials, EMG onset latency was significantly affected by group (unimpaired vs. stroke: F_(1,18)_ = 9.43, p = 0.0066) and muscle type (F_(1,790)_ = 322.48, p<0.0001), as well as the interaction of these factors (F_(1,790)_ = 68.45, p<0.0001). During voluntary flexion, the average onset latency of the Br muscle activity was 147±57 ms for unimpaired subjects and 226±80 ms for stroke subjects, a statistically significant difference (Figure 3C-solid columns, p = 0.04). Conversely during startReact flexion, EMG onset latency was not significantly affected by group (F_(1,18)_ = 1.09; p = 0.3092), but was affected by muscle type (F_(1,161)_ = 59.08; p<0.0001). The interaction of these factors was not significant (F_(1,161)_ = 1.82; p = 0.1782). Post-hoc comparisons demonstrated that during startReact movements, there was no significant difference between Br onset latencies between unimpaired (101±42 ms) and stroke (85±32 ms) populations (Figure 3C-dashed columns, p = 0.18).

The pattern of muscle activity (timing of agonist/antagonist firing) of stroke and unimpaired subjects was significantly different during voluntary flexion but not different during startReact flexion. The relative timing between the agonist and antagonist onset latencies was not significantly different between stroke and unimpaired subjects during the startReact flexion movements (stroke: 37±21 ms, unimpaired: 32±33 ms; p = 0.15). This same measure differed substantially between groups during the voluntary flexion movements (stroke: 154±86 ms, unimpaired: 56±55 ms; p<0.001).

### Voluntary and startReact during extension trials

**Figure 4:**
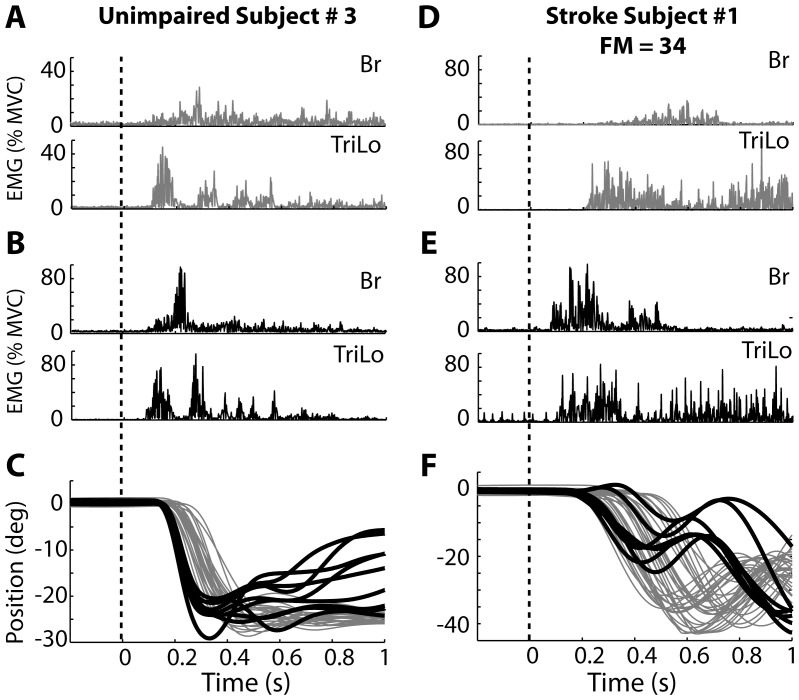
Sample data from typical unimpaired and stroke subjects during extension. (A, B, C) Data from unimpaired subject. (D, E, F) Data from stroke subject. (A, D) EMG responses in the Br and TriLo muscles during voluntary reaching. (B, E) EMG responses during startReact reaching. (C,F) Elbow position during voluntary (gray) and startReact (black) reaching.

The results of the extension trials were similar to those of the flexion trials for the unimpaired subjects. Muscle activity during voluntary trials (Figure 4A) was initiated slower than during the startReact trials (Figure 4B). The patterns of muscle activity were similar in both conditions, with the agonist (TriLo) leading the antagonist (Br). EMG amplitude was increased in both muscles during the startReact conditions. The decreased onset latency and increased muscle activation resulted in faster movement initiation, as measured by the change in elbow position (Figure 4C).

**Figure 5:**
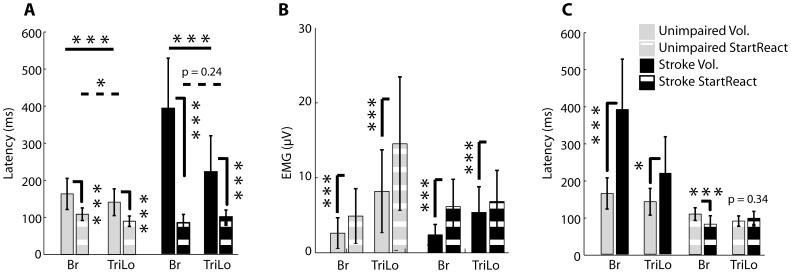
Group **Results** **for extension task.** (A) Comparison of voluntary and startReact reaching latencies in unimpaired and stroke subjects. (B) Comparison of voluntary and startReact mean EMG amplitudes in unimpaired and stroke subjects. (C) Comparison between reaching latencies of unimpaired and stroke subjects during voluntary and startReact reaching. Same data are presented as in (A), reorganized to facilitate comparison. Means and standard deviations are presented above. Stars indicate significance: * = p-value<.05, ** = p-value<.01, and *** = p-value<.001.

These results were consistent across all unimpaired subjects. EMG onset latency was significantly affected by condition (F_(1,510)_ = 136.28, p<0.0001) and muscle type (F_(1,510)_ = 32.51, p<0.0001), though there was no significant interaction between these factors (F_(1,510)_ = 0.10, p = 0.7475). The agonist, TriLo muscle, was activated at 141+/−36 ms and 90+/−14 ms during voluntary and startReact extension, respectively, a significant difference (p<0.0001). The antagonist, Br muscle, latency was activated at 164+/−42 ms and 109+/−42 during voluntary and startReact extension respectively, again a significant difference (p<0.0001). Appropriate patterns of muscle activity were maintained, with the TriLo leading Br during voluntary (p<0.0001) and startReact (p<0.03) extension (Figure 5A, gray bars). EMG amplitude was affected by condition (F_(1,510)_ = 128.95, p<0.0001) and muscle type (F_(1,510)_ = 343.51, p<0.0001) with a significant interaction (F_(1,510)_ = 38.17, p<0.0001). EMG amplitude during startReact extension trials (Br = 4.9+/−3.6 µV, TriLo = 14+/−8.9 µV) was significantly higher in both the Br (p<0.0001) and TriLo (p<0.0001) muscles compared to voluntary extension amplitudes (Br = 2.6+/−2.0 µV, TriLo = 8.2+/−5.5 µV) in unimpaired individuals (Figure 5B; gray bars).

In the stroke group, startReact extension was initiated faster than voluntary extension and had increased muscle activity, but the patterns of muscle activity differed from those observed during the voluntary trials. Voluntary extension movements following stroke were initiated slower than those in unimpaired subjects, but exhibited similar patterns of muscle activation, with the agonist leading the antagonist (Figure 4D, gray). The onset of muscle activity was faster during the startReact trials, but the task-appropriate phasing of the agonist and antagonist muscles was not preserved in stroke subjects (Figure 4E). Often, activity in the antagonist (Br) led that in the agonist (TriLo). This inappropriate activation pattern could be observed in the corresponding elbow trajectories, which often moved towards flexion (up) before moving toward the task appropriate direction of extension (down).

The inappropriate phasing of the agonist and antagonist muscle activity during the startReact extension trials was observed in all stroke participants. EMG onset latencies were significantly influenced by condition (F_(1,439)_ = 409.55; p<0.0001) and muscle type (F_(1,439)_ = 213.40; p<0.0001), as well as their interaction (F_(1,439)_ = 77.55; p<0.0001). Br and TriLo muscle onset latencies during voluntary extension (Br = 390+/−136, TriLo = 219+/−98) were significantly slower (Br: p<0.0001; TriLo: p<0.0001) than during startReact extension (Br = 81+/−22, TriLo = 98+/−18). However, appropriate pattern of muscle activity (TriLo leading Br) was not present during startReact extension. Specifically, there was no difference between the latency of Br and TriLo (p = 0.24) (Figure 5A, black bars). In contrast, voluntary extension trials of stroke subjects had appropriate activation with the agonist (TriLo) leading the antagonist (Br) (p<0.0001). EMG amplitude was affected by condition (F_(1,439)_ = 62.64 p<0.0001) and muscle type (F_(1,439)_ = 164.25, p<0.0001) with a significant interaction (F_(1,439)_ = 8.19, p = 0.0044). It was found that muscle activity was increased in both the Br (p<0.0001) and TriLo (p<0.0001) muscles during startReact extension (Br = 3.66.2+/−, TriLo = 6.8+/−4.1) compared to voluntary extension extension (Br = 2.4+/−1.7, TriLo = 5.4+/−3.4) (Figure 5B, black bars).

Though unimpaired subjects initiated voluntary movements significantly faster than stroke subjects, the onset latency of the agonist, TriLo, muscle was not different between the two populations during startReact extension. During voluntary movement, the average onset latency was significantly affected by the subject group (F_(1,17)_ = 15.45, p = 0.0011) and muscle type (F_(1,752)_ = 299.16, p<0.0001), as well as their interaction (F_(1,752)_ = 181.90, p<0.0001). The voluntary latency of the TriLo muscle was 141±36 ms in unimpaired subjects and 219±97 ms in stroke subjects, a significant difference (p = 0.02). In contrast to the voluntary extension trials, the main effects of subject group and muscle type were not significant during startReact extension, though their interaction was (subject group: F_(1,17)_ = 1.95, p = 0.1798; muscle type: F_(1,180)_ = 2.39, p = 0.1235; interaction: F_(1,180)_ = 32.11; p<0.0001). There was no difference found between the onsets of the TriLo muscle in unimpaired subjects (90±14 ms) and stroke subjects (97±18 ms) during startReact extension (p = 0.34). Corresponding to its early onset latency, activation of the Br (antagonist) muscle was significantly faster in stroke subjects (81±22 ms) than unimpaired subjects (109±17 ms) during the extension startReact trials (Figure 5C, p<0.0001).

### Startle in the absence of a movement plan

**Figure 6:**
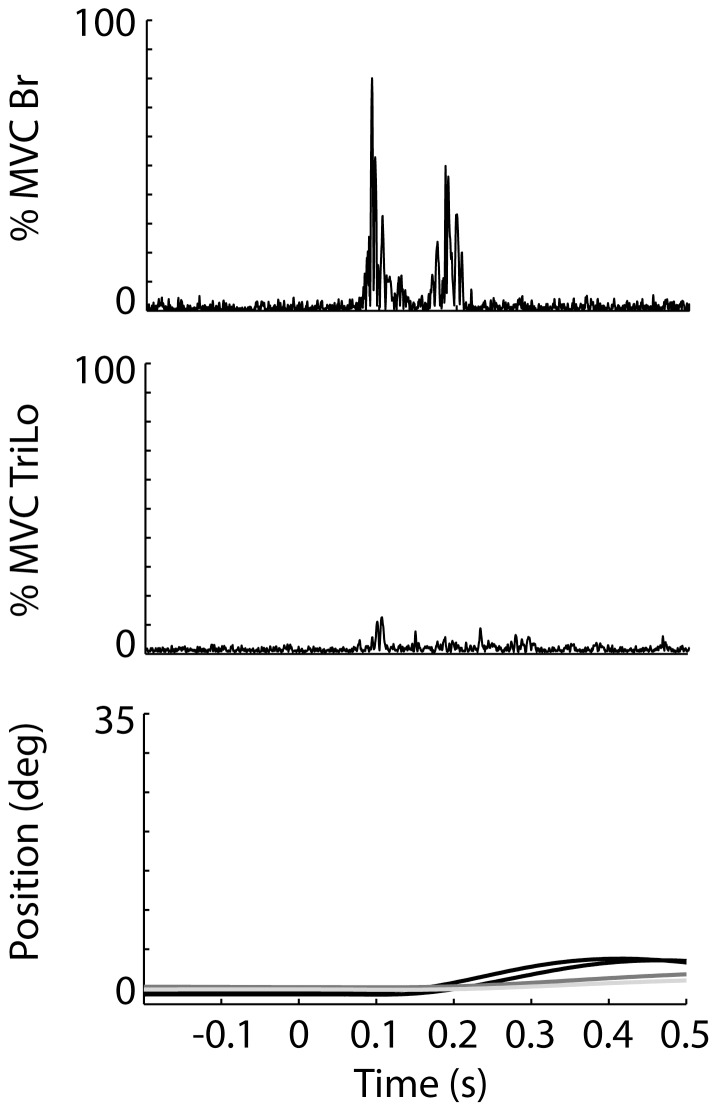
Startle in the absence of a movement plan. EMG activity from the Br (top) and TriLo (middle) muscles from the first startle (absence of a movement plan) trial for stroke subject # 7 (FM = 22). Elbow position for all startle trials (C).

Startle in the absence of a movement plan (i.e. classic startle) in both unimpaired and stroke subjects typically resulted in no deviation from the HOME position, or in a small movement in the flexion direction (Figure 6). Most unimpaired subjects responded with no movement (N = 5) or with small movements of less than 8° (N = 2). Two unimpaired subjects responded with intermittent large deviations (25°) away from HOME, but these were always were in the direction of the movement TARGET and likely corresponded to the subject planning a movement prior to the WARNING cue. Similarly, most stroke subjects responded with no movement (N = 4) or small flexion movements of less than 8° (N = 3). Only two subjects responded with large deviations from the HOME position. In both cases, these large movements were made in the flexion direction. No stroke subjects responded to startle at the WARNING signal with an extension movement.

Classic startle was quantified further in stroke subjects. The onset latency was significantly affected by muscle type (F_(1,111)_ = 27.53, p = <0.0001) but not trial type (F_(2,111)_ = 0.46, p = 0.63). During classic startle, the latency of Br and TriLo muscle activation were not different (p = 0.79). Comparisons between muscle activation onsets of classic startle and startReact flexion showed a difference between TriLo muscle onset latencies (0.02) but not Br muscle onset latencies (0.36). Comparisons between classic startle and startReact extension show no differences in onset latencies between Br (0.79) and TriLo (0.08) muscles.

### Adaptation of abnormal flexor activity in startReact extension

**Figure 7:**
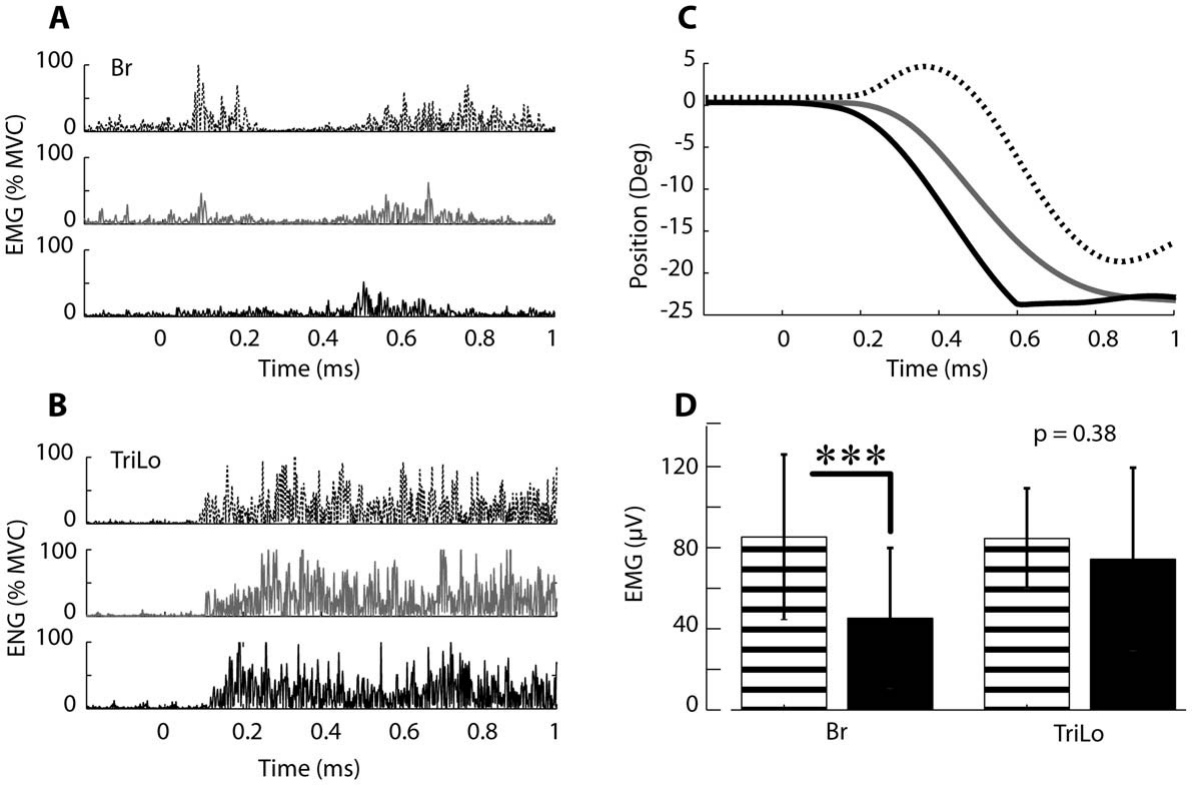
Adaptation of startReact extension movements. EMG activity in the Br (A) and TriLo (B) muscles during the first (dashed) middle (gray) and last (black) startReact trials collected from Subject #9 (FM = 40). Elbow position during the first, middle, and last trials (C). Group statistical results comparing the mean EMG amplitude during the first (dashed) and last (black) trial for the Br and TriLo muscles.

The inappropriate activity in the flexors during the startReact extension trials decreased over time. The early onset of activity in the Br muscle was largest in the first startReact extension trial, slowly decreasing in amplitude across successive trials (Figure 7A). This effect was not present in the agonist muscle, TriLo, which had a consistent level of activity across startReact trials (Figure 7B). This alteration in muscle activation amplitude resulted in increasingly appropriate elbow movements with successive trials (Figure 7C). The adaptation in the muscle activation amplitude was quantified by comparing the EMG amplitudes from the first and last startReact extension trials from all subjects (Figure 7D). The Br muscle activation was significantly lower during the last startReact trial compared to the first (p<0.0001), while TriLo muscle showed no difference (p = 0.38) (Figure 7D). Adaptation in the Br muscle was consistent in all subjects, and present regardless of impairment level, although we were unable to quantify adaptation in our most severely impaired individual (subject 6; FM = 10), since only 2 startReact trials could be collected due to fatigue.

### Fugl-Meyer score vs. muscle activation latency in voluntary and startReact movements

**Figure 8:**
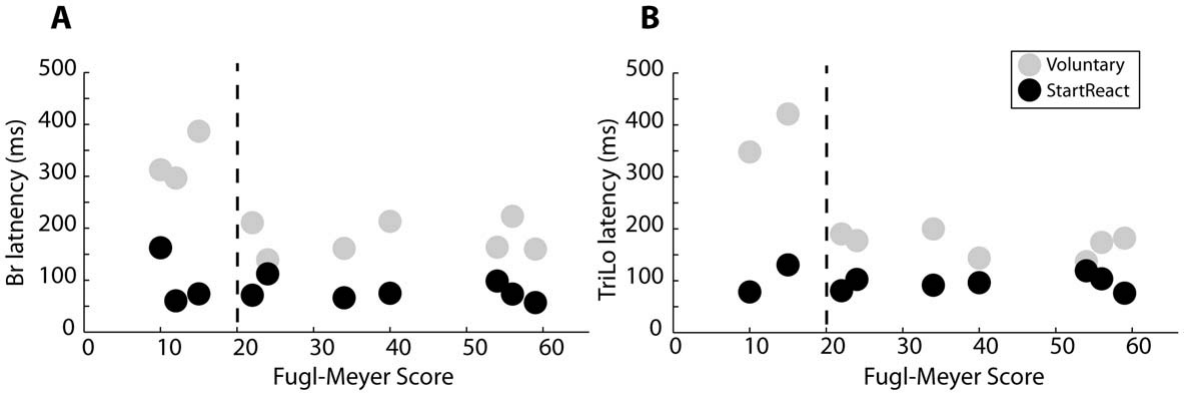
Fugl-Meyer score vs muscle activation latency. (A) The latency of agonist muscle firing during voluntary and startReact flexion task graphed against Fugl-Meyer score of stroke subjects. (B) Latency of agonist muscle firing during extension tasks presented as in (A).

Although impairment level, as assessed by the Fugl-Meyer score, had a substantial effect on the agonist latency during voluntary movement, it had no corresponding effect on the agonist latency during startReact movements (Figure 8). The most impaired stroke subjects (Fugl-Meyer<20), had voluntary latencies significantly slower than the less impaired stroke subjects in extension (Δ = 212 ms, p = <0.001) and flexion (Δ = 150 ms, p = <0.001). This difference was not present for startReact movement, for which all subjects had similar latencies in extension (Δ = 8 ms, p = 0.59) and flexion (Δ = 19 ms, p = 0.42).

## Discussion

### Summary

The objective of this study was to quantify the behavior of the startReact response following stroke, and to use this behavior to assess the ability to plan appropriate elbow movements. We found that stroke subjects possess an intact startReact response that allowed them to initiate movements as rapidly as unimpaired individuals. The muscle coordination patterns were not statistically different between stroke and unimpaired individuals during startReact flexion tasks. In other words when flexion movements were initiated involuntarily, movements were not different between unimpaired and stroke subjects. This is striking given the differences in the voluntary movements of these two groups, most specifically the slower initiation latency and poor muscle coordination patterns of stroke subjects. As the startReact response results in the involuntary release of a planned movement, our result suggests that stroke subjects may be planning more appropriate movements than they can release voluntarily. The results during extension movements were less straightforward for our stroke subjects. Task-inappropriate flexor activity interrupted startReact elbow extension in stroke subjects. Still, this inappropriate activity diminished over time while the task-appropriate activity in the agonist muscle remained steady. This adaptation suggests that the inappropriate activity was transient in nature and not related to the underlying movement plan. We hypothesize that the task-inappropriate flexor activity during extension results from an inability to suppress the classic startle reflex, which primarily influences flexor muscles and adapts rapidly with successive stimuli. Such an interpretation is consistent with the proposal that startle and startReact are mediated by separate neural structures [19], [20].

### Movement planning following stroke

StartReact flexion movements in stroke subjects were remarkably similar to those elicited in unimpaired subjects. Despite the significantly slower and less coordinated muscle activity during voluntary movements, startReact allowed stroke subjects to initiate elbow flexion movements as quickly as unimpaired individuals and with muscle coordination patterns that were not different between the groups. Importantly, startReact flexion movements in stroke subjects were distinctive from the classic startle response in this same group, indicating that classic startle alone is not responsible for the observed muscle coordination. The classic startle response occurs when a subject, not preparing to move, is presented with a loud startling acoustic stimulus. In our experiment, the stimulus was presented at the WARNING cue before subjects were asked to prepare to move. Under these conditions (i.e. the classic startle response), the startling acoustic stimulus elicited synchronous firing of the Br and TriLo muscles (p = 0.79) and small (<8 degree) elbow flexion movements in most stroke subjects. This is in contrast to the muscle activation observed during startReact flexion where the Br and TriLo muscles were activated at different latencies (p = 0.01). StartReact flexion also generated large elbow movements that were sufficient to reach the target position (25 degrees) 82% of the time. A final demonstration of the distinctive patterns of muscle activity was showcased by the different onset latency of TriLo during classic startle and the startReact flexion tasks (p = 0.02). These results establish that the EMG patterns observed between classic startle and startReact flexion are distinctive in stroke subjects demonstrating that the muscle activity during startReact flexion is the release of a prepared movement.

The literature indicates that agonist and antagonist bursts are planned together [21], [22]. If so, these results indicate that stroke survivors retain the capacity to plan coordinated muscle activation patterns that are similar to those in unimpaired individuals, at least with regard to the simple single-joint movements considered in this study. In addition, the startReact response enhances the amplitude of agonist muscular activity. Together, these results suggest startReact provides a means to execute faster, stronger, and more appropriate movements than stroke subjects can elicit voluntarily.

The results during extension movements were less straightforward for our stroke subjects. As in flexion, startReact extension movements were initiated as quickly as unimpaired individuals. Similarly, the amplitude of the agonist EMG was enhanced during startReact. Unlike startReact flexion, startReact extension exhibited task-inappropriate flexor (antagonist) activity that often led extensor (agonist) activity. This activity resulted in elbow flexion preceding elbow extension for some trials. Interestingly, this inappropriate activity diminished over time while the task-appropriate agonist activity remained steady. While possible, the time course of these experiments makes it unlikely that the adaptation resulted from a training effect. The transient nature of the task-inappropriate activity, which differs from the steady nature of the agonist activity suggest that it is not related to the underlying movement plan. This led us to hypothesize that the task-inappropriate flexor activity results from an inability to suppress the classic startle reflex during the startReact response.

The task-inappropriate flexor activity during the startReact extension trials shares many qualities with the classic startle response. First, classic startle and startReact extension both result in synchronous firing in Br and TriLo muscles. Second, the classic startle response, like the abnormal flexor activity, adapts quickly over time [4]. The notion that startle could interrupt startReact extension is supported by evidence suggesting that startle and startReact are two separate phenomena [19], [20]. Kumru et al. (2006) demonstrated that they could selectively inhibit the classic startle response while maintaining startReact. Alibiglou et al. (2012) showcased that classic startle and startReact may utilize different neural pathways for expression. Since the cortex is known to modulate the amplitude of classic startle [23], we hypothesize that following a stroke the cortex loses ability to inhibit the classic startle response during startReact movements leading to simultaneous expression of both. This would be analogous to other instances of unsuppressed reflexes following stroke, like spasticity (hypermetric stretch reflex) [24], [25], [26], [27] and the resurgence of the typically dormant asymmetric tonic neck reflex [28], [29]. While the classic startle reflex adapts over time, startReact does not. This explains why the inappropriate flexor activity (hypothesized classic startle) showed adaptation but the agonist extensor activity (startReact) remained constant. It is probable that this same phenomenon occurs during startReact flexion but as extensor activity during classic startle is small (or absent) it does not substantially disrupt the startReact flexion movement.

While we cannot quantify the startReact response in isolation from the classic startle reflex, we would expect an intact elbow extension movement plan. The fact that 9 of 10 stroke subjects exhibited movements that ultimately went in the extension direction indicates that an extension plan was present. If no plan was present, we would expect elbow movements similar to the classic startle response (small flexion movements <8 degrees) as opposed to the extension movements our stroke subject exhibited. None of our stroke subjects performed elbow extension movements when tested for classic startle.

### Neural pathways relevant to startReact following stroke

The presence of the classic startle reflex indicates that the neural structures responsible for this phenomenon remain at least partially intact following stroke. The pathways mediating the startle reflex have been extensively studied in animal preparations, most recent evidence points to a simple circuit mediated through the pontomedullary reticular formation [30], [31]. Expression of the startle reflex does not require the cortex, as evidenced by its remaining functionality in decerebrate animals [32]. Our results showing a functional startle reflex indicate that the brainstem circuit mediating the startle reflex remains intact in the stroke subjects tested. This is significant because the brainstem is known to contribute to motor tasks such as reaching [33], [34], balance [35], [36], [37], [38], and locomotion [39], [40], [41], [42]. Therefore if we can tap into this remaining functional circuitry, we may be able to improve not only the effectiveness of arm movements but also ambulation.

An intact startReact response requires not only the appropriate initiation circuitry, currently theorized to be the same as that required for the classic startle reflex [31], but also an intact movement plan. Movement planning is a global, distributive event shared by many layers of the nervous system [33], [34], [43], [44]. The cortical regions most strongly linked to movement planning are the primary, premotor, and supplementary motor cortices, known to have strong projections to the reticular formation [45], [46], [47], [48], also strongly linked to movement planning [33], [34]. Given the impairments of our stroke population, it is unlikely that all of these cortical structures were intact. Still, the planning of ballistic elbow movements remained functional. This suggests that there is enough redundancy in the nervous system to effectively compensate for cortical loss following stroke, or that additional structures, such as the regions of the tectum that contribute to rapid coordination of eye and hand movements [49], are significantly involved in the planning process. Without specific lesion data, it is not possible to differentiate between these two hypotheses. We anticipate that lesions to cortical and subcortical (including basal ganglia) regions will differentially impair movement planning following stroke. Careful evaluation of lesion characteristics and startReact deficits may provide an opportunity to isolate the differential roles of these regions, all known to be involved movement planning. Finally, it is important to note that these results pertain to single-joint elbow movements and more research is needed to determine if our results on movement planning extend to more complex tasks involving multiple joints or fine movements of the fingers, which require more extensive use of the cortex.

Our results that faster, stronger, and more appropriate movements can be elicited by startReact suggests that impaired voluntary initiation and execution of movement is a dominant factor contributing to movement impairment following stroke. There are a number of well documented deficits in neuromotor physiology that could contribute to execution impairments following stroke. Cortical damage leads to substantial remapping of the cortical motor areas, including an increased overlap of regions for neighboring joints and an increased involvement of ipsilateral projections [50]. There is also significant loss of corticospinal tracts [44], known to be critically involved in voluntary movements [51], [52] and long-latency reflex responses [53]. Together, these changes likely contribute to the decreased ability to activate individual muscles and joints [54] and to the delayed activation of muscles [1]. Furthermore during movement execution, there is continuous feedback from proprioceptive systems. This feedback is also impaired following stroke [55], [56], [57], [58], contributing to spasticity and muscle discoordination. Updating even an appropriately planned movement with inappropriate feedback could contribute to the inability to execute the intended movements following stroke.

### Functional significance

Our results demonstrate that stroke subjects have the capacity to appropriately plan ballistic elbow movements and to release them as quickly as unimpaired individuals. These results suggest that therapies focused on enhancing movement execution are appropriate, and that these therapies might benefit from employing alternate methods to trigger planned movements during the training process, like startReact responses. Further studies are necessary to determine whether the startReact phenomenon can be utilized as a therapy tool.

Second, our results also highlight a new deficit; the inappropriate flexor activity, which leads to deflection away from the intended target during startReact extension movements. A link has been established between quick corrective responses following perturbations of the arm and whole body and startReact [59] [60], [61]. Cohesively, these observations suggest that stroke subjects may respond to startling disturbances with movements in the wrong direction. Such a mechanism may contribute to the increased risk of falling that is prevalent in stroke subjects [62].
